# Automated machine learning to predict the difficulty for endoscopic resection of gastric gastrointestinal stromal tumor

**DOI:** 10.3389/fonc.2023.1190987

**Published:** 2023-05-10

**Authors:** Luojie Liu, Rufa Zhang, Dongtao Shi, Rui Li, Qinghua Wang, Yunfu Feng, Fenying Lu, Yang Zong, Xiaodan Xu

**Affiliations:** ^1^ Department of Gastroenterology, Changshu Hospital Affiliated to Soochow University, Suzhou, China; ^2^ Department of Gastroenterology, The First Affiliated Hospital of Soochow University, Suzhou, China; ^3^ Department of Gastroenterology, No.1 People’s Hospital of Kunshan, Suzhou, China; ^4^ Department of Gastroenterology, No.2 People’s Hospital of Changshu, Suzhou, China; ^5^ Department of General Surgery, Changshu Hospital Affiliated to Soochow University, Suzhou, China

**Keywords:** automated machine learning, predictive models, endoscopic resection, gastrointestinal stromal tumors, difficulty

## Abstract

**Background:**

Accurate preoperative assessment of surgical difficulty is crucial to the success of the surgery and patient safety. This study aimed to evaluate the difficulty for endoscopic resection (ER) of gastric gastrointestinal stromal tumors (gGISTs) using multiple machine learning (ML) algorithms.

**Methods:**

From December 2010 to December 2022, 555 patients with gGISTs in multi-centers were retrospectively studied and assigned to a training, validation, and test cohort. A *difficult case* was defined as meeting one of the following criteria: an operative time ≥ 90 min, severe intraoperative bleeding, or conversion to laparoscopic resection. Five types of algorithms were employed in building models, including traditional logistic regression (LR) and automated machine learning (AutoML) analysis (gradient boost machine (GBM), deep neural net (DL), generalized linear model (GLM), and default random forest (DRF)). We assessed the performance of the models using the areas under the receiver operating characteristic curves (AUC), the calibration curve, and the decision curve analysis (DCA) based on LR, as well as feature importance, SHapley Additive exPlanation (SHAP) Plots and Local Interpretable Model Agnostic Explanation (LIME) based on AutoML.

**Results:**

The GBM model outperformed other models with an AUC of 0.894 in the validation and 0.791 in the test cohorts. Furthermore, the GBM model achieved the highest accuracy among these AutoML models, with 0.935 and 0.911 in the validation and test cohorts, respectively. In addition, it was found that tumor size and endoscopists’ experience were the most prominent features that significantly impacted the AutoML model’s performance in predicting the difficulty for ER of gGISTs.

**Conclusion:**

The AutoML model based on the GBM algorithm can accurately predict the difficulty for ER of gGISTs before surgery.

## Introduction

Gastric gastrointestinal stromal tumors (gGISTs) are the most common mesenchymal tumors of the gastrointestinal tract (1). Endoscopic resection (ER) is a minimally invasive and effective treatment option for small GISTs, but the procedure can be challenging for larger and more complex tumors (2, 3). To ensure the safety and efficacy of ER, it is essential to predict the difficulty of the procedure beforehand accurately. Traditional methods of predicting difficulty rely on subjective assessment by experienced endoscopists, which can be influenced by interobserver variability and other factors. Su et al. (4) have made the first-ever prediction of the difficulty in ER of gGISTs by constructing a nomogram. The area under the receiver operating characteristic (ROC) curves (AUC) and the accuracy of this model in predicting surgical difficulty were found to be 0.756 and 0.798, respectively. Although the model has demonstrated exemplary performance, finer models could yield even better results.

Machine learning (ML) is becoming increasingly prevalent in medicine because of its efficient computing algorithms, which enable the learning of valuable insights from vast amounts of clinical data (5, 6). Previous studies (7–11) have established the immense potential of ML in developing models for disease diagnosis, predicting prognosis, analyzing survival rates, and other medical applications. Automated machine learning (AutoML), a new type of ML, intelligently chooses from a range of algorithms and hyperparameters to create customized models based on specific target data (12, 13). Compared to traditional ML, AutoML utilizes intelligent early stopping, regularization, hyperparameter optimization, and cross-validation techniques, allowing for the development of more accurate models in less time.

In this study, we aimed to provide a dataset consisting of clinical and endoscopic features of patients with gGISTs from multiple centers. We used this dataset to train, validate, and test a series of machine learning models to predict the difficulty for ER of gGISTs.

## Material and methods

### Patients

We conducted a retrospective analysis of consecutive patients who underwent ER of gGISTs at the First Affiliated Hospital of Soochow University between December 2010 and December 2022. The patients were randomly divided into training and validation cohorts in a 7:3 ratio. In addition, we gathered information on patients who received ER of gGISTs at Changshu Hospital Affiliated to Soochow University, No.1 People’s Hospital of Kunshan, and No.2 People’s Hospital of Changshu from January 2017 to December 2022. This data was used to create the test cohort for the study. The main inclusion criteria were (1): diagnosis of gGIST through pathological and immunohistochemical examination after surgery (2); regular preoperative blood routine, coagulation tests, and electrocardiogram results (3); absence of lymph node or distant metastasis in patients. Patients who met any of the following criteria were excluded from the study (1): lesions with a high risk of malignancy based on EUS evaluation (2); patients with synchronous lesions in multiple locations (3); patients with multiple lesions in the stomach (4); patients with poor cardiopulmonary function and unable to undergo anesthesia and surgery (5); incomplete medical records of the patient. Our institutions received ethical approval for the clinical research study protocol from the ethics committee. Before the ER procedure, all patients were thoroughly informed about the advantages and potential risks and provided with a signed written consent form. The reporting of this study conforms to STROBE guidelines (14).

### Endoscopic equipment and procedures

Based on the nature of the lesion, we employed three distinct ER techniques: endoscopic submucosal dissection (ESD), endoscopic full-thickness resection (EFTR), and submucosal tunnel endoscopic resection (STER). ESD is employed to treat gGISTs that arise from either the muscularis mucosae (MM) or muscularis propria (MP) and protrude into the lumen. If GISTs originate from the deep MP with extraluminal growth or tumors that cannot be separated from the serosal layer during ESD, EFTR can be utilized as a treatment. STER is mainly used for gGISTs that grow in the gastroesophageal junction or greater curvature of the stomach, where a submucosal tunnel can be quickly established. Comprehensive information regarding ER procedures can be found in the previous publication (15–17). Although the endoscopists involved in the procedures had varying degrees of experience with ER of gGISTs, all cases were performed by senior endoscopists with extensive experience. These endoscopists had previously completed over 5,000 gastroscopy and colonoscopy procedures and more than 200 EMR procedures for gastrointestinal polyps before performing ER for gGISTs. In our study, an endoscopist was considered experienced in ER of gGISTs once he or she had carried out a cumulative sum (CUSUM) of 50 such procedures. General anesthesia and endotracheal intubation were administered to all patients. All patients were placed in the left lateral position. The ER procedures utilized either a dual knife (KD-650L; Olympus^®^, Japan), an insulated-tip knife (KD-611L; Olympus^®^, Japan), or a combination of the two. A single-channel endoscope (GIF-Q260J, Olympus^®^, Japan) equipped with a transparent cap on its tip was employed. The energy output was achieved using a High-frequency electric coagulation and electrocautery device (ERBE^®^ VIO 200D). Other equipment utilized during the procedures included metallic clips, nylon loops (LeClampTM^®^ Loop-20 and Loop-30; Leo, Changzhou, China), over-the-scope clips (OTSC), injection needles, hot biopsy forceps, and a carbon dioxide insufflator.

### Postoperative management

Following surgery, specimens were preserved in a 10% formalin solution, and immunohistochemical staining (including CD117, CD34, and Dog-1, among others) was conducted to confirm the diagnosis. Typically, patients receive nasogastric decompression after surgery to prevent postoperative complications. They are instructed to fast for two days, or three days or more in the case of EFTR patients, depending on their postoperative status. Blood routine, CRP, and/or calcitonin tests were carried out after surgery, and all patients were administered proton pump inhibitors, gastric mucosal protective agents, nutritional support, and fluid replacement. When patients exhibited abdominal pain or muscle tension, a CT or orthostatic X-ray scan was performed to rule out postoperative perforation. Antibiotic therapy or surgical treatment was administered based on their condition. For patients who experienced intraoperative perforation or postoperative infection, antibiotics were prescribed.

### Data collection

Patient information, such as gender, age, history of smoking or alcohol consumption, primary symptoms, medical history, American Society of Anesthesiologists (ASA) score (18), body mass index (BMI), tumor size, location, shape, depth of invasion, boundary characteristics, procedure duration, intraoperative and postoperative complications, R0 resection rates, ER technique used, modified National Institutes of Health (NIH) risk criteria (19), number of days of postoperative fasting, and length of hospital stay following surgery, were gathered from electronic medical records of our institutions.

### Definitions

A difficult case was defined as meeting one of the following criteria: an operative time ≥ 90 min, severe intraoperative bleeding, or conversion to laparoscopic resection. The operative time was determined from the point at which the submucosal injection began to the completion of the closure of the defect. The origin of the tumor was identified based on preoperative endoscopic ultrasonography (EUS) examination. Tumors with a round, oval, or nodular shape were categorized as having a regular shape, whereas those with a branching shape were designated as having an irregular shape. Severe intraoperative bleeding was characterized by repeated endoscopic hemostasis, a postoperative decrease in hemoglobin levels exceeding 2 g/dL, or necessitating surgical assistance (20, 21). Tumor characteristics, such as tumor size and location, were assessed based on preoperative endoscopic ultrasound examination or abdominal-enhanced computed tomography (CT) scans. Postoperative complications included delayed bleeding, delayed perforation, and postoperative infection. Delayed bleeding was defined as clinical evidence of bleeding that occurred after ER, as evidenced by hematemesis or melena, a decline in hemoglobin levels of more than 2.0 g/dL within 24 hours, or the need for endoscopic therapy (22). Delayed perforation was verified through X-ray or CT. Postoperative infection was determined by a postoperative body temperature exceeding 37.5°C and/or an increase in inflammatory indicators such as blood routine, CRP, or calcitonin (23). R0 resection was defined as the surgical removal of a tumor with no residual cancerous tissue detected in the margins of the excised tissue, as confirmed by histological examination of the specimen’s radial and deep margins (24).

### Automated machine learning

AutoML analysis was carried out using the H2O package installed from the H2O.ai platform (www.h2o.ai), which automatically selects and combines suitable algorithms into several ensemble models. The set of algorithms comprises a randomized grid of Gradient Boosting Machines (GBMs), a randomized grid of Deep Neural Networks (DLs), a default Random Forest (DRF), and a fixed grid of Generalized Linear Models (GLMs). Hyperparameter optimization was conducted through a 5-fold cross-validation grid search on the training set, where various combinations of hyperparameters included in the grid search were evaluated based on their AUCs. AutoML visualization was presented through feature importance, SHapley Additive exPlanation (SHAP), and Local Interpretable Model Agnostic Explanation (LIME) techniques. Through SHAP analysis, it was possible to determine the key features that significantly influenced the model predictions and the extent of their contribution to the overall model performance for a specific prediction (25). By randomly selecting examples from the test set, LIME analysis illustrated the contribution of each feature toward predicting the outcome (26).

### Statistical analysis

Categorical variables were expressed as frequencies and percentages, and the Chi-square test or Fisher exact test was used to compare groups. Continuous variables were expressed as the median and interquartile ranges (IQR), and a comparison between the two groups was made using the Mann-Whitney U test. To address the issue of multiple collinear relationships among the explanatory variables, a univariate analysis was performed using the least absolute shrinkage and selection operator (LASSO) regression model with the minimum criterion. The model was then further refined using a binary logistic backward stepwise regression analysis. The predictive performance of the resulting model was evaluated using the areas under the receiver operating characteristic curves (AUC), calibration curve, and decision curve analysis (DCA). Furthermore, a nomogram was constructed based on the independent risk factors identified in the multivariate analysis. The statistical significance level was set at *P* < 0.05. R software (version 4.1.0) was utilized for conducting all the statistical analyses.

## Results

### Baseline characteristics of patients and lesions

**Figure 1:**
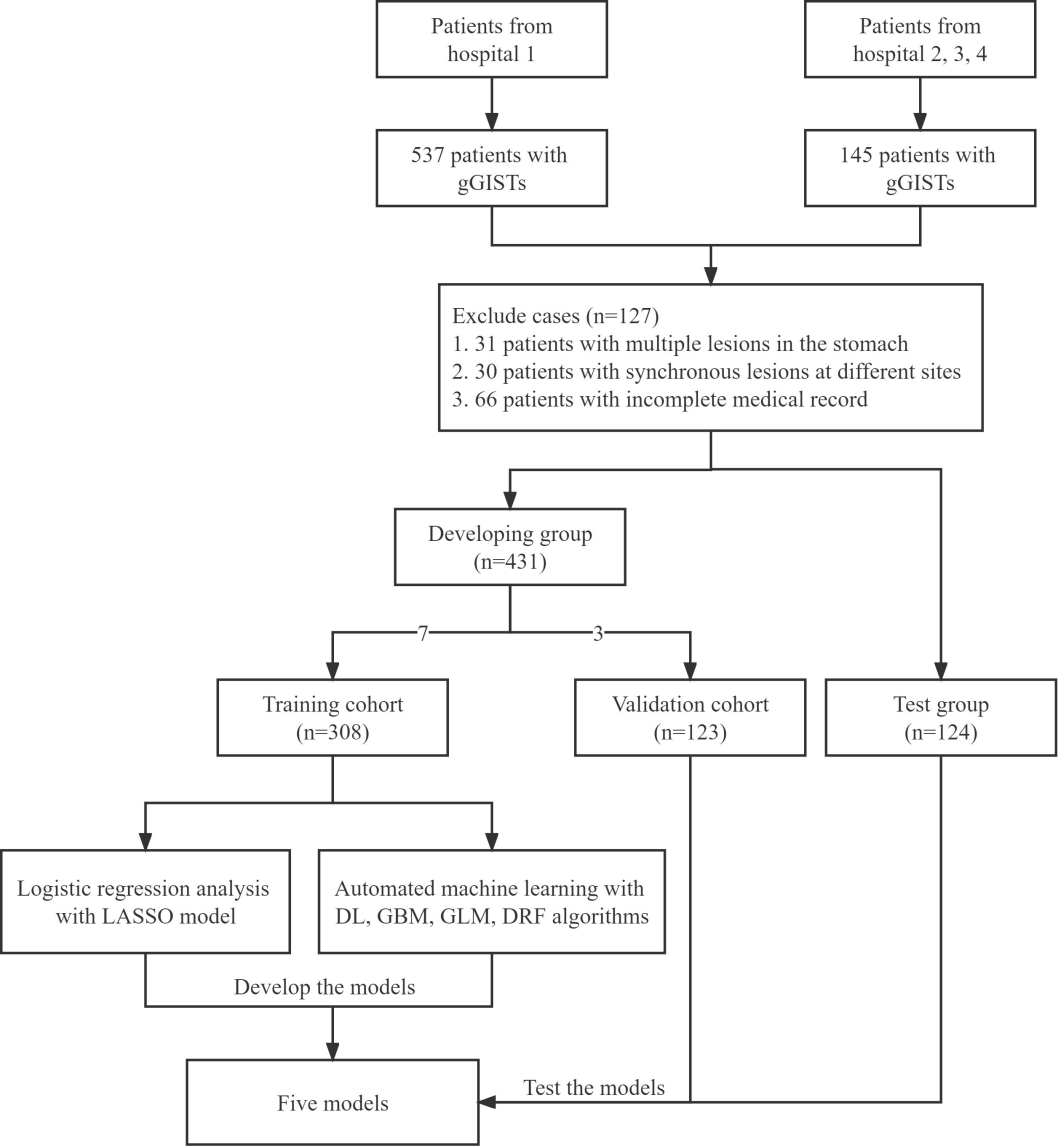
Flow chart of the study. gGISTs, gastric gastrointestinal stromal tumors; GBM, gradient boost machine; DL, deep neural net; GLM, generalized linear model; DRF, default random forset; LASSO, least absolute shrinkage and selection operator.

In this study, a total of 555 patients were enrolled, out of which 97 cases (17.5%) experienced difficulty in the whole cohort. **Figure 1** illustrates the study protocol in the form of a flow chart, while **Table 1** presents the features of 555 gGISTs in the developing and test cohorts. In the developing dataset, there were 195 men (45.2%) and 236 women (54.8%). The proportion of patients aged < 60 years in the difficult group was 43.0%, while in the non-difficult group, it was 51.7%. In the test dataset, the proportion of female patients with gGISTs is higher than that of male patients (62.9% *vs*. 37.1%). No significant differences were observed between the two groups of three datasets in terms of sex, age, history of smoking or alcohol consumption, medical history, ASA score, and BMI (*P* > 0.05).

**Table 1:** Demographic and clinical characteristics of patients in training, validation and test groups.

Variables	The developing dataset (n=431)			The test dataset (n=124)		
	Difficulty (n=79)	Non-difficulty (n=352)	*P*-value	Difficulty (n=18)	Non-difficulty (n=106)	*P*-value
Gender, n (%)			0.118			0.485
Male	42 (53.2)	153 (43.5)		8 (44.4)	38 (35.8)	
Female	37 (46.8)	199 (56.5)		10 (55.6)	68 (64.2)	
Age, yesrs, n (%)			0.164			0.093
< 60	34 (43.0)	182 (51.7)		12 (66.7)	48 (45.3)	
≥ 60	45 (57.0)	170 (48.3)		6 (33.3)	58 (54.7)	
Primary symptom, n (%)			0.501			0.028*
Asymptomatic	16 (20.3)	73 (20.7)		4 (22.2)	26 (24.5)	
Abdominal discomfort	60 (75.9)	273 (77.6)		12 (66.7)	80 (75.5)	
Hemorrhage	3 (3.8)	6 (1.7)		2 (11.1)	0	
Smoking, n (%)			0.828			0.775
Yes	25 (31.6)	107 (30.4)		5 (27.8)	33 (31.1)	
No	54 (68.4)	245 (69.6)		13 (72.2)	73 (68.9)	
History of drinking, n (%)			0.836			0.969
Yes	16 (20.3)	75 (21.3)		4 (22.2)	24 (22.6)	
No	63 (79.7)	277 (78.7)		14 (77.8)	82 (77.4)	
Hypertension, n (%)			0.649			0.916
Yes	27 (34.2)	111 (31.5)		6 (33.3)	34 (32.1)	
No	52 (65.8)	241 (68.5)		12 (67.7)	72 (67.9)	
Coronary disease, n (%)			0.168			0.824
Yes	21 (26.6)	69 (19.6)		3 (16.7)	20 (18.9)	
No	58 (73.4)	283 (80.4)		15 (83.3)	86 (81.1)	
Diabetes, n (%)			0.096			0.768
Yes	26 (32.9)	84 (23.9)		4 (22.2)	27 (25.5)	
No	53 (67.1)	268 (76.1)		14 (77.8)	79 (74.5)	
ASA score, n (%)			0.693*			1.000*
I	64 (81.0)	292 (83.0)		16 (88.9)	90 (84.9)	
II	15 (19.0)	59 (16.8)		2 (11.1)	16 (15.1)	
III	0	1 (0.3)		0	0	
BMI, kg/m², n (%)			0.143			0.368
< 18.5	19 (24.1)	57 (16.2)		6 (33.3)	20 (18.9)	
18.5-23.9	39 (49.4)	169 (48.0)		8 (44.4)	54 (50.9)	
≥ 24.0	21 (26.6)	126 (35.8)		4 (22.2)	32 (30.2)	
Location 1, n (%)			**<0.001**			0.115
Upper	71 (89.9)	235 (66.8)		14 (77.8)	60 (56.6)	
Middle	5 (6.3)	79 (22.4)		2 (11.1)	38 (35.8)	
Lower	3 (3.8)	38 (10.8)		2 (11.1)	8 (7.5)	
Location 2, n (%)			0.843			1.000
Lesser curvature	30 (38.0)	118 (33.5)		5 (27.8)	27 (25.5)	
Greater curvature	5 (6.3)	30 (8.5)		2 (11.1)	11 (10.4)	
Anterior	30 (38.0)	141 (40.1)		7 (38.9)	44 (41.5)	
Posterior	14 (17.7)	63 (17.9)		4 (22.2)	24 (22.6)	
Shape, n (%)			**<0.001**			0.693
Regular	59 (74.7)	324 (92.0)		15 (83.3)	92 (86.8)	
Irregular	20 (25.3)	28 (8.0)		3 (16.7)	14 (13.2)	
Invasion depth, n (%)			**<0.001**			**0.001**
MP (within)	52 (65.8)	313 (88.9)		10 (55.6)	92 (86.8)	
MP-ex	27 (34.2)	39 (11.1)		8 (44.4)	14 (13.2)	
Boundary, n (%)			**0.037**			0.707
Clear	64 (81.0)	315 (89.5)		14 (77.8)	78 (73.6)	
Unclear	15 (19.0)	37 (10.5)		4 (22.2)	28 (26.4)	
Size, cm, n (%)			**<0.001**			**<0.001**
≥ 3.0	36 (45.6)	29 (8.2)		6 (33.3)	4 (3.8)	
2.0-3.0	25 (31.6)	90 (25.6)		8 (44.4)	16 (15.1)	
< 2.0	18 (22.8)	233 (66.2)		4 (22.2)	86 (81.1)	
Experience, cases, n (%)			**<0.001**			**0.005**
< 50	46 (58.2)	97 (27.6)		12 (66.7)	34 (32.1)	
≥ 50	33 (41.8)	255 (72.4)		6 (33.3)	72 (67.9)	
Endoscopic tecnique, n (%)			0.068*			0.266*
ESD	30 (38.0)	182 (51.7)		6 (33.3)	56 (52.8)	
EFTR	48 (60.8)	164 (46.6)		12 (66.7)	48 (45.3)	
STER	1 (1.3)	6 (1.7)		0	2 (1.9)	
Modified NIH risk criteria, n (%)			<0.001*			<0.001*
Very low	30 (38.0)	271 (77.0)		2 (11.1)	84 (79.2)	
Low	25 (31.6)	63 (17.9)		8 (44.4)	16 (15.1)	
Intermediate	19 (24.1)	17 (4.8)		8 (44.4)	4 (3.8)	
High	5 (6.3)	1 (0.3)		0	2 (1.9)	
Operative time, min, median (IQR)	105.0 (95.0,124.0)	52.0 (41.0,64.0)	**<0.001**	95.0 (90.0,102.5)	60.0 (50.0,72.0)	**<0.001**
Conversion, n (%)	14 (17.7)	0	<0.001*	0	0	NA
Severe intraoperative bleeding, n (%)	14 (17.7)	0	<0.001*	4 (22.2)	0	**<0.001**
Postoperative hospitalization, days,	6.0 (5.0,8.0)	6.0 (5.0,6.0)	**<0.001**	7.0 (6.0,8.3)	5.0 (4.0,6.0)	**<0.001**
median (IQR)						
Postoperative fasting, days,	3.0 (3.0,5.0)	3.0 (2.0,3.0)	**<0.001**	3.0 (2.8,5.3)	2.0 (2.0,3.0)	**<0.001**
median (IQR)						
R0 resection, n (%)	55 (69.6)	339 (96.3)	**<0.001**	12 (66/7)	96 (90.6)	**0.005**
Postoperative complications, n (%)	21 (26.6)	37 (10.5)	**<0.001**	10 (55.6)	6 (5.7)	**<0.001**

ASA, American Society of Anesthesiologists; BMI, body mass index; MP, muscularis propria; MP-ex, MP with exophytic growth; ESD, endoscopic submucosal dissection; EFTR, endoscopic full-thickness resection; STER, submucosal tunnel endoscopic resection; NIH, National Institute of Health; IQR, interquartile ranges; *Fisher’s exact test; “NA” means no statistical analysis was performed.

### Univariate and multivariate logistic regression analysis

**Figure 2:**
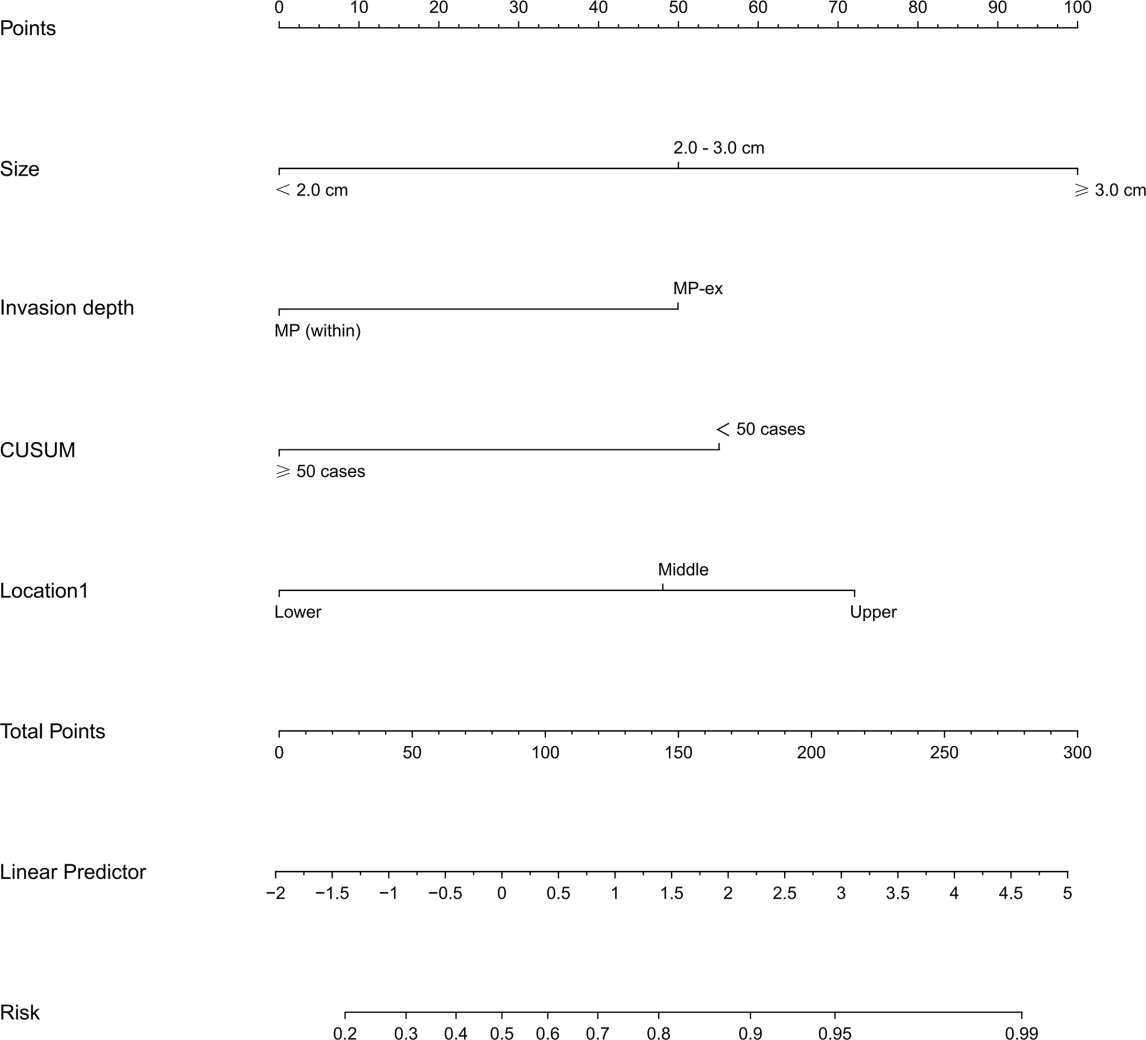
Nomogram of the LASSO model for predicting the difficulty for endoscopic resection of gGIST. LASSO, least absolute shrinkage and selection operator; gGISTs, gastric gastrointestinal stromal tumors.

By utilizing the LASSO regression model with a minimum criterion attained through 5-fold cross-validation, four variables out of 17 were selected and designated as independent risk factors. This approach was employed to address the issue of multiple collinear relationships among the explanatory variables, as depicted in **Supplementary Figure 1**. A logistic model comprising of four variables (tumor size, invasion depth, location, and endoscopists’ experience) was ultimately established and presented as both a nomogram and a score system, suitable for clinical utilization (**Figure 2**). The calibration curves pertaining to the training set, validation set, and test set are depicted in **Supplementary Figure 2**, and the mean absolute errors being 0.021, 0.035 and 0.043, respectively. The calibration curves provided evidence that the LASSO model’s estimated risk was in close proximity to the actual risk, implying a considerable level of dependability. The DCA plots of the LASSO model in the test set demonstrated that when the threshold probability of a difficult procedure predicted by the LASSO model was between 20% and 100%, an intervention might add more benefit (10% - 80%) (**Supplementary Figure 3**). The DCA plots of the AutoML models are presented in **Supplementary Figure 4**, and the net benefit of these models is about 80%.

### Automated machine learning analysis

**Figure 3:**
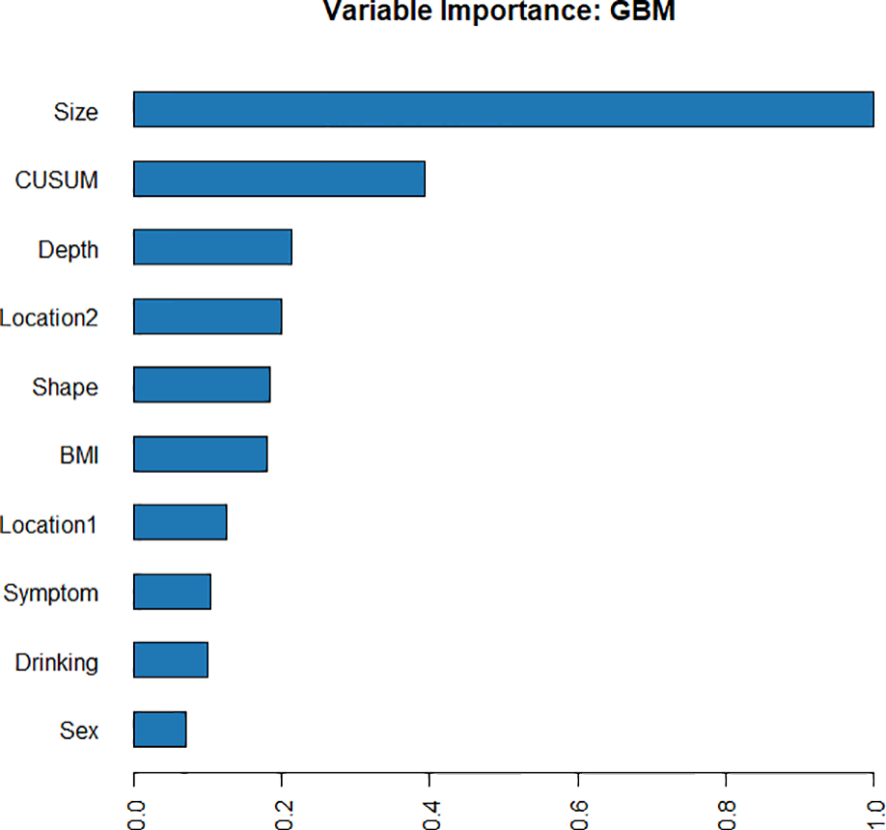
Variable importance of the GBM model in the training cohort, showing that tumor size was the most important feature, followed by endoscopists’ experience (CUSUM), invasion depth, etc.

**Figure 4:**
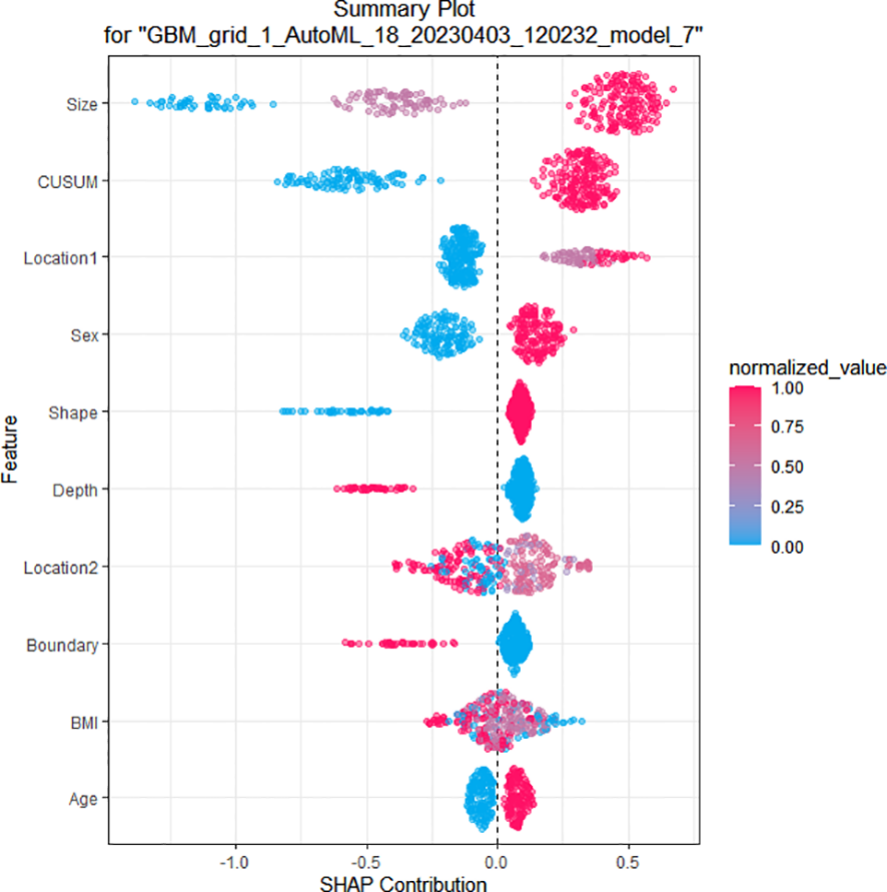
SHAP of the GBM model in the training cohort. As a variable’s value approaches 1, the likelihood of a patient having a difficult procedure increases. SHAP, SHapley Additive exPlanation; GBM, gradient boost machine.

**Figure 5:**
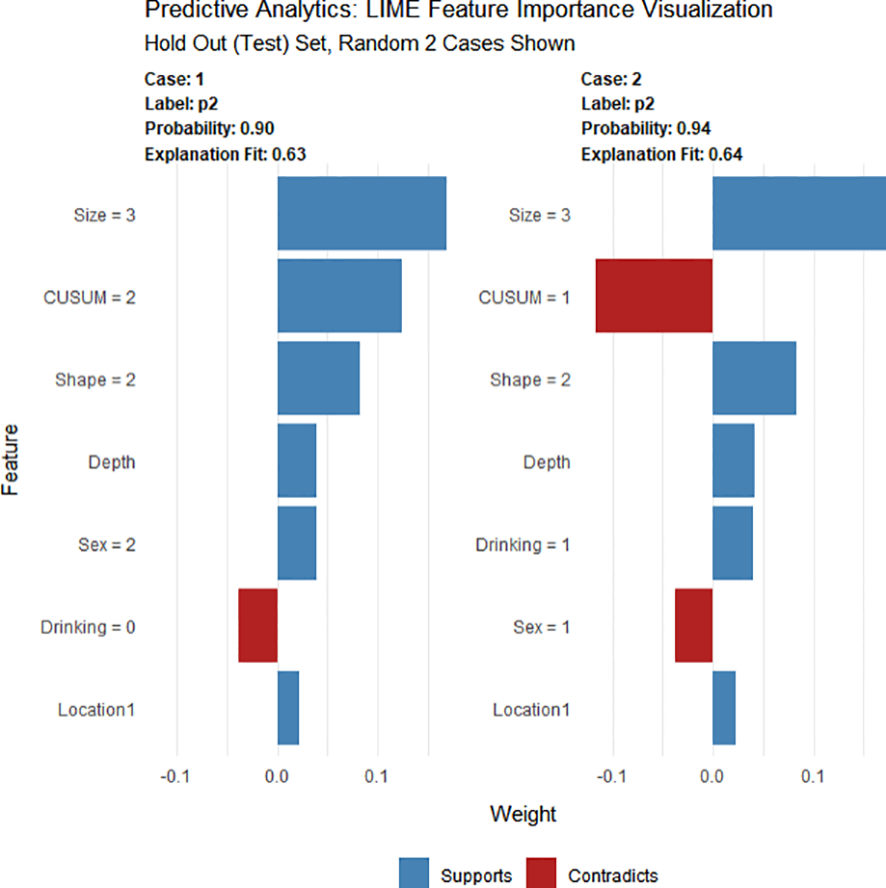
LIME of the GBM model in the test cohort. LIME, Local Interpretable Model Agnostic Explanation.

Using four ML algorithms (GBM, DL, GLM, and DRF), 64 models were constructed, with the stacked ensemble models being excluded due to limited interpretability. The GBM model outperformed the other models, exhibiting the highest AUC values and accuracy, and consequently deemed the most optimal model. **Figure 3** indicates that tumor size was identified as the most crucial feature, followed by endoscopists’ experience, invasion depth, location (cross-sectional), shape, BMI, location (longitudinal), primary symptom, history of smoking, and sex, in that order of importance. Additionally, tumor size, endoscopists’ experience, invasion depth, and location (longitudinal) were identified as the common important variables shared by the GBM and logistic regression models. **Figure 4** displays the SHAP contribution plots generated by GBM algorithms, illustrating the ten most significant variables, namely tumor size, endoscopists’ experience, location (cross-sectional), sex, shape, invasion depth, location (longitudinal), boundary, BMI, and age. As a variable’s value approaches 1, the likelihood of a patient having a difficult procedure increases. For example, the red dots in the SHAP plot corresponding to tumors ≥ 3.0cm are predominantly located on the right side of the zero axis, indicating that patients with tumors larger than 3.0cm are more likely to experience a difficult procedure. As shown in **Table 2**, the GBM algorithm outperformed the DL, DRF, and GLM algorithms in the validation cohort regarding AUC, with a higher value of 0.894 compared to 0.881, 0.858, and 0.854, respectively. Furthermore, the accuracy values for the GBM algorithm were the highest compared to the DL, DRF, and GLM algorithms, with 0.935, 0.870, 0.854, and 0.878, respectively. Among these 5 models, the DRF model has the highest sensitivity, with values of 1.000 in both the validation and test sets, but the lowest specificity, with values of 0.847 and 0.862, respectively. The LASSO model has the lowest sensitivity in both the validation and test sets, with values of 0.739 and 0.556, respectively. The DL and GLM models have intermediate performance in terms of AUC, sensitivity, specificity, and accuracy among these models. A LIME plot based on the GBM model for the test cohort showcased the impact of various significant variables on the difficulty for ER of gGISTs. For example, based on the GBM model, **Figure 5** demonstrates that case 2 had a predicted probability of 0.94 for experiencing a difficult procedure. Tumor size greater than 3.0cm was identified as the most critical predictor for difficult procedures, followed by irregular tumor shape, invasion depth beyond MP, history of alcohol consumption, and tumor location in the upper third of the stomach. Conversely, the effect of the experienced endoscopist and male gender had a mitigating effect on these factors.

**Table 2:** Comparison of AutoML models and logistic regression analysis in predicting the difficulty for ER of gGISTs in the validation cohort.

	AUC	Sensitivity	Specificity	Accuracy	PPV	NPV	LR+	LR-
**AutoML**								
** GBM**	0.894	0.917	0.937	0.935	0.611	0.990	14.536	0.089
** DL**	0.881	0.769	0.882	0.870	0.435	0.970	6.509	0.262
** DRF**	0.858	1.000	0.847	0.854	0.217	1.000	6.556	0
** GLM**	0.854	0.900	0.876	0.878	0.391	0.990	7.264	0.114
**Logistic regression analysis**								
** LASSO**	0.835	0.739	0.930	0.894	0.708	0.939	10.559	0.281

ER, endoscopic resection; gGIST, gastric gastrointestinal stromal tumor; AutoML, automated machine learning; PPV, positive predictive value; NPV, negative predictive value; LR+, positive likelihood ration; LR-, negative likelihood ration; AUC, areas under the receiver operating characteristic curves; GBM, gradient boost machine; DL, deep neural net; DRF, default random forest; GLM, generalized linear model; LASSO, least absolute shrinkage and selection operator.

## Discussion

This study aimed to evaluate the difficulty for ER of gGISTs using multiple ML algorithms. A total of 555 patients with gGISTs were retrospectively studied and assigned to a training, validation, and test cohort. Five algorithms were employed in building models, and the GBM model outperformed other models with an AUC of 0.894 in the validation cohort and 0.791 in the test cohort. The AutoML model based on the GBM algorithm can accurately predict the difficulty for ER of gGISTs before surgery, and tumor size and endoscopists’ experience were identified as the most prominent features that significantly impacted the performance of the AutoML model. This study provides a machine learning-based approach for accurately predicting the surgical difficulty for ER of gGISTs.

Accurate preoperative assessment of surgical difficulty is crucial to the success of the surgery and patient safety. By predicting the difficulty of the surgical procedure before surgery, surgeons can better prepare for the surgery, optimize the surgical plan, and ensure patient safety during the operation (27, 28). Su et al. (4) are the only ones who have predicted the difficulty for ER of gGISTs so far. Their study defined a *difficult procedure* as an operative time greater than 90 minutes or severe intraoperative bleeding. However, previous studies have suggested that conversion to laparoscopic or open surgery indicates difficult surgery (29–31) because it may increase operative time, blood loss, and postoperative recovery time, thereby increasing the risk to patients. Therefore, meeting one of the following criteria was used to define a difficult case in this study: operative time of 90 minutes or more, severe bleeding during the surgery, or the need to convert to laparoscopic resection or open surgery.

The SHAP analysis revealed that in our study, the most crucial feature of the GBM model is tumor size. The result agreed with the findings of the logistic regression model in our study and aligned with the risk factors for the endoscopic surgical difficulty reported in the literature (4, 32). According to the studies by Su et al. (4) and Jian et al. (32), ER was challenging for tumors larger than 3.0 cm in size. In treating gGISTs with larger tumor sizes, the limited operating space in the ER results in poorer functional space and surgical field of view. Consequently, endoscopists must frequently adjust the angle of the endoscopic incision and the volume of air in the stomach cavity to achieve complete tumor removal. Therefore, for gGISTs with larger tumor sizes, ER should be performed by experienced endoscopists, as this study found that surgical experience is also an essential factor affecting the difficulty of the procedure. Experienced endoscopists may have better technical proficiency and higher success rates, enabling them to adapt better to the surgical environment, accomplish surgical tasks more effectively, and reduce the incidence of surgical complications. Sun et al. (33) reported that the learning curve for ER of gastric submucosal tumors was approximately 32 cases, while Yoshida et al. (34) retrospectively analyzed the learning curve of 7 novice endoscopists in ER of gastric lesions and found that a stable state could be reached after completing around 30 cases. To account for potential variations in the learning curves of different endoscopists, a minimum threshold of 50 GIST excisions was established in this study to ensure that the endoscopists had adequate experience conducting ER for gGISTs. In this study, we divided tumor size into three groups: < 2.0cm, 2.0-3.0cm, and ≥ 3.0cm, and endoscopists’ experience into <50 cases and ≥50 cases. The larger the tumor, and the less experience the endoscopist has, the more difficult the surgery becomes. Therefore, we recommend that endoscopists lacking surgical experience should choose lesions with smaller diameters for surgical intervention.

We utilized five different ML algorithms to construct predictive models with high accuracy. Our models achieved superior AUC and accuracy compared to the nomogram model built by Su et al. (4). Furthermore, by accurately assessing the surgical difficulty for ER of gGISTs, this study can assist doctors in understanding potential challenges prior to surgery, thereby improving the success rate of the operation and patient safety. Additionally, this multi-center research boasts a larger sample size and higher external validity and reduces potential biases caused by the unique circumstances of a single research center. However, our study had some limitations. First, our study may have had selection bias and information bias due to its retrospective nature. Future research could use a prospective study design to more accurately evaluate the effectiveness of different ML algorithms in predicting the difficulty for ER of gGISTs. Second, this study did not consider the postoperative prognosis and patient recovery. Future research could incorporate these factors to comprehensively evaluate the clinical application value of ML models in predicting the difficulty for ER of gGISTs. Third, the advancements in novel medical devices and surgical techniques may affect the difficulty of ER, and the factors influencing surgical difficulty may also change. Therefore, it is crucial to keep pace with the latest developments when studying the difficulty for ER of gGISTs. Fourth, due to differences in procedural steps, the difficulty levels of ESD, EFTR, and STER endoscopic techniques may vary. Conducting more in-depth research on individual endoscopic techniques could aid in identifying and analyzing the specific difficulties associated with each technique. Fifth, due to the low prevalence of gGISTs, our validation cohort consisted of only 123 cases and the test cohort included 124 cases. Adding more samples later would be better.

In conclusion, our study evaluated the difficulty for ER of gGISTs using ML algorithms. The GBM model outperformed others, achieving high accuracy in predicting ER difficulty. Tumor size and endoscopists’ experience were identified as influential factors. The GBM-based AutoML model shows promise for preoperative assessment, but further validation on diverse datasets and consideration of new medical technologies are needed to enhance its clinical applicability.

## Data availability statement

The raw data supporting the conclusions of this article will be made available by the authors, without undue reservation.

## Ethics statement

The studies involving human participants were reviewed and approved by the ethics committee of the first affiliated hospital of Soochow University. The patients/participants provided their written informed consent to participate in this study.

## Author contributions

LL and RZ contributed equally to this work. All authors contributed to the article and approved the submitted version.

## Supplementary material

Supplementary Figure 1 A chart showing the penalties for predictive factors indicating the level of difficulty for endoscopic resection of gGISTs was derived through LASSO regression analysis. Left: Regression coefficients. With the value of λ increasing, the absolute values of coefficients decrease. Right: Identification of the optimal λ value in the LASSO regression analysis was achieved by 5-fold cross-validation. (The left vertical line is drawn using the minimum criterion and the right vertical line is drawn using the 1_se criterion. In our study, LASSO regression model with minimum criterion was used in the univariate analysis in order to solve such multiple co-linear relationships among the explanatory variables. LASSO: least absolute shrinkage and selection operator; gGIST, gastric gastrointestinal stromal tumors.

Supplementary Figure 2 Calibration curve of the LASSO model in the training, validation and test set, with the mean absolute errors being 0.021, 0.035 and 0.043, respectively.

Supplementary Figure 3 Decision curve analysis of the LASSO model in the test set. The DCA plots demonstrated that when the threshold probability of a difficult procedure predicted by the LASSO model was between 20% and 100%, an intervention might add more benefit (10% - 80%).

Supplementary Figure 4 Decision curve analysis plots of 4 AutoML models in the test set, indicating net benefits of around 80%. **(A)** DL model; **(B)** GBM model; **(C)** GLM model; **(D)** DRF model.
