# Supplementary figures and images for: Automated machine learning to predict the difficulty for endoscopic resection of gastric gastrointestinal stromal tumor
Source: Front Oncol. 2023 May 10;13:1190987. doi: 10.3389/fonc.2023.1190987 (PMC10206233; doi:10.3389/fonc.2023.1190987)

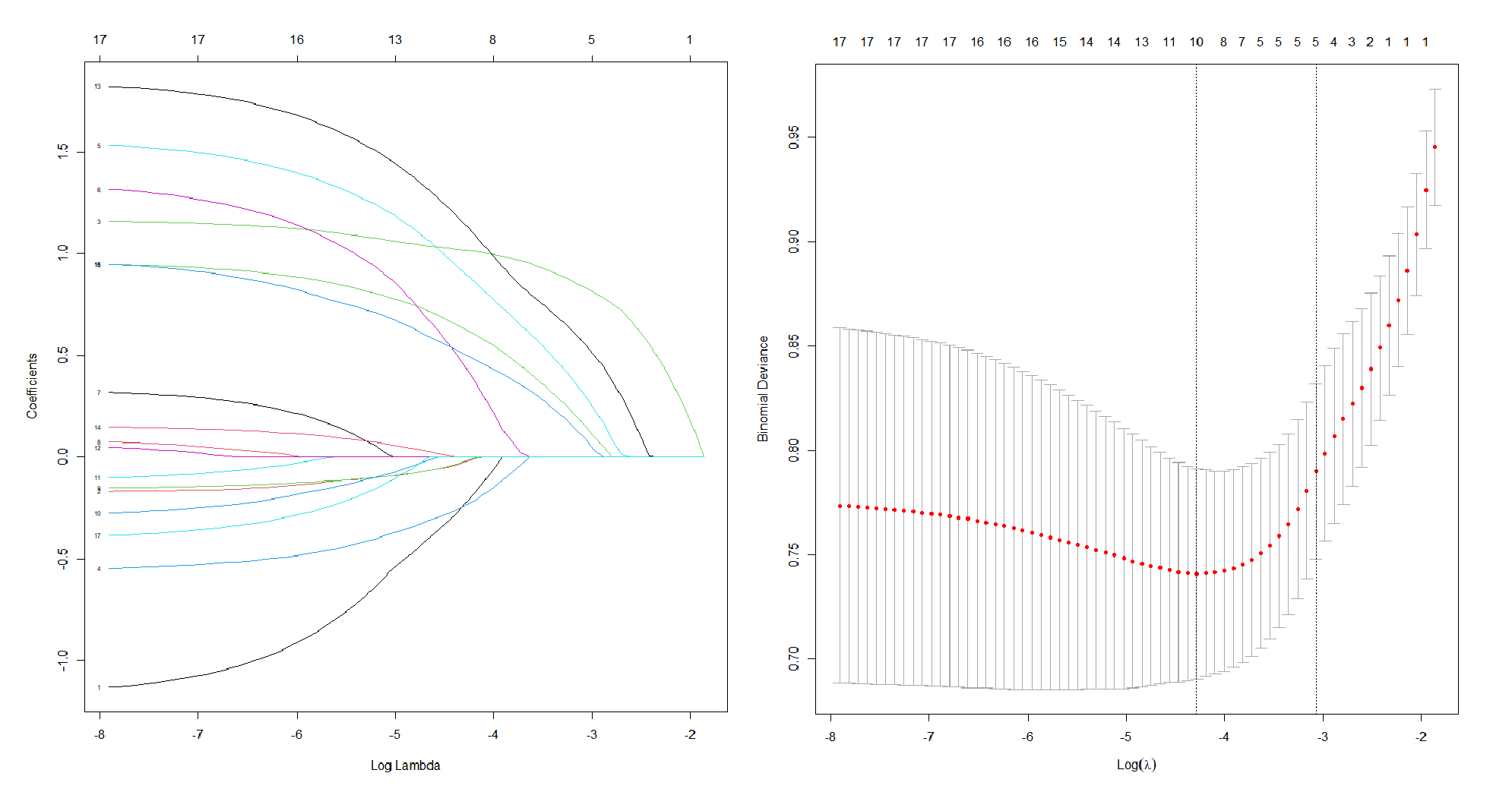

Supplement: Supplementary Figure 1 — A chart showing the penalties for predictive factors indicating the level of difficulty for endoscopic resection of gGISTs was derived through LASSO regression analysis. Left: Regression coefficients. With the value of λ increasing, the absolute values of coefficients decrease. Right: Identification of the optimal λ value in the LASSO regression analysis was achieved by 5-fold cross-validation. (The left vertical line is drawn using the minimum criterion and the right vertical line is drawn using the 1_se criterion. In our study, LASSO regression model with minimum criterion was used in the univariate analysis in order to solve such multiple co-linear relationships among the explanatory variables. LASSO: least absolute shrinkage and selection operator; gGIST, gastric gastrointestinal stromal tumors. [file Image_1.tif]

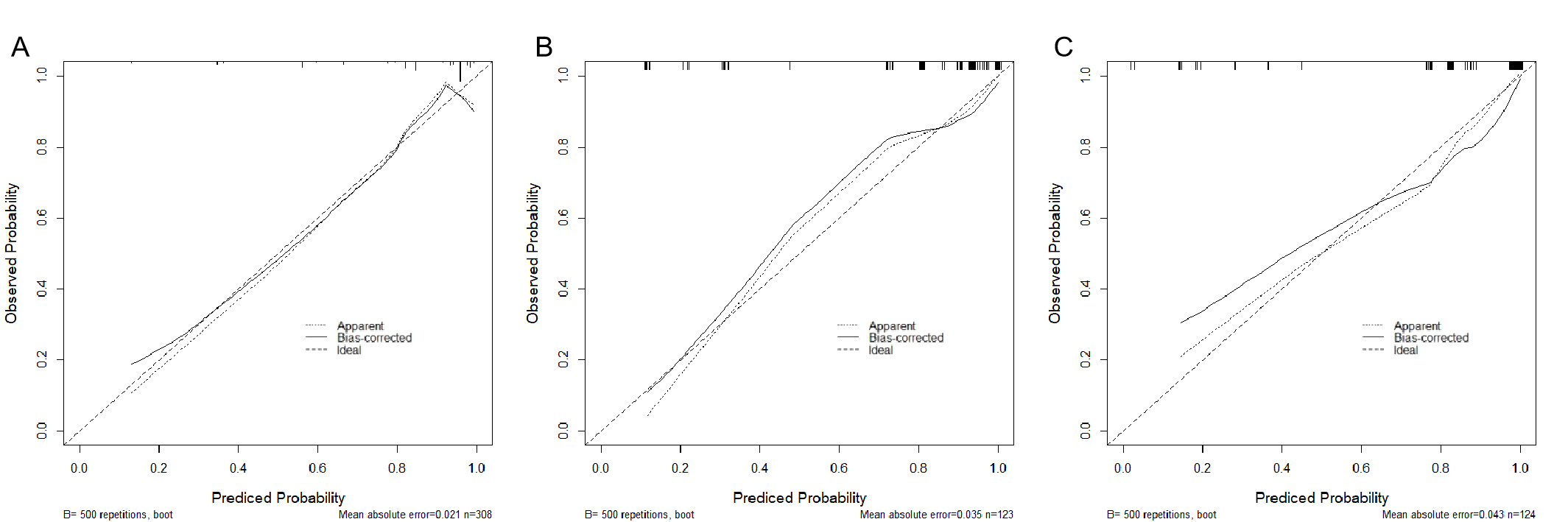

Supplement: Supplementary Figure 2 — Calibration curve of the LASSO model in the training, validation and test set, with the mean absolute errors being 0.021, 0.035 and 0.043, respectively. [file Image_2.tif]

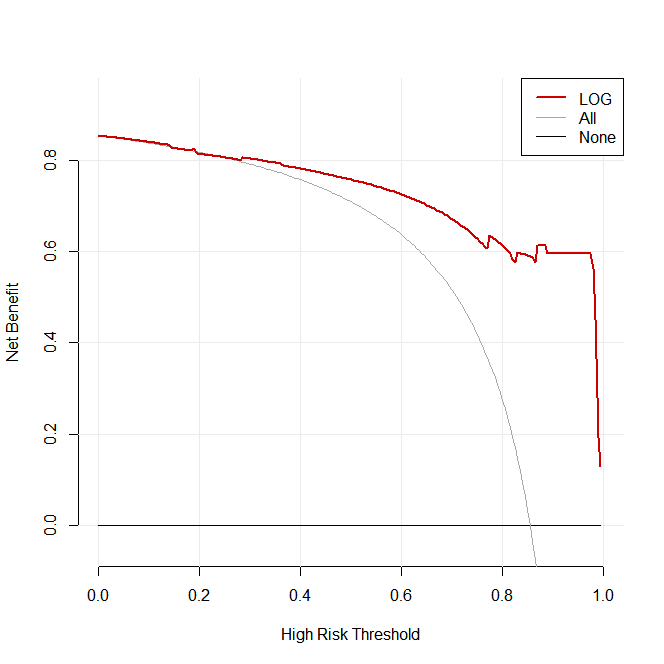

Supplement: Supplementary Figure 3 — Decision curve analysis of the LASSO model in the test set. The DCA plots demonstrated that when the threshold probability of a difficult procedure predicted by the LASSO model was between 20% and 100%, an intervention might add more benefit (10% - 80%). [file Image_3.tiff]

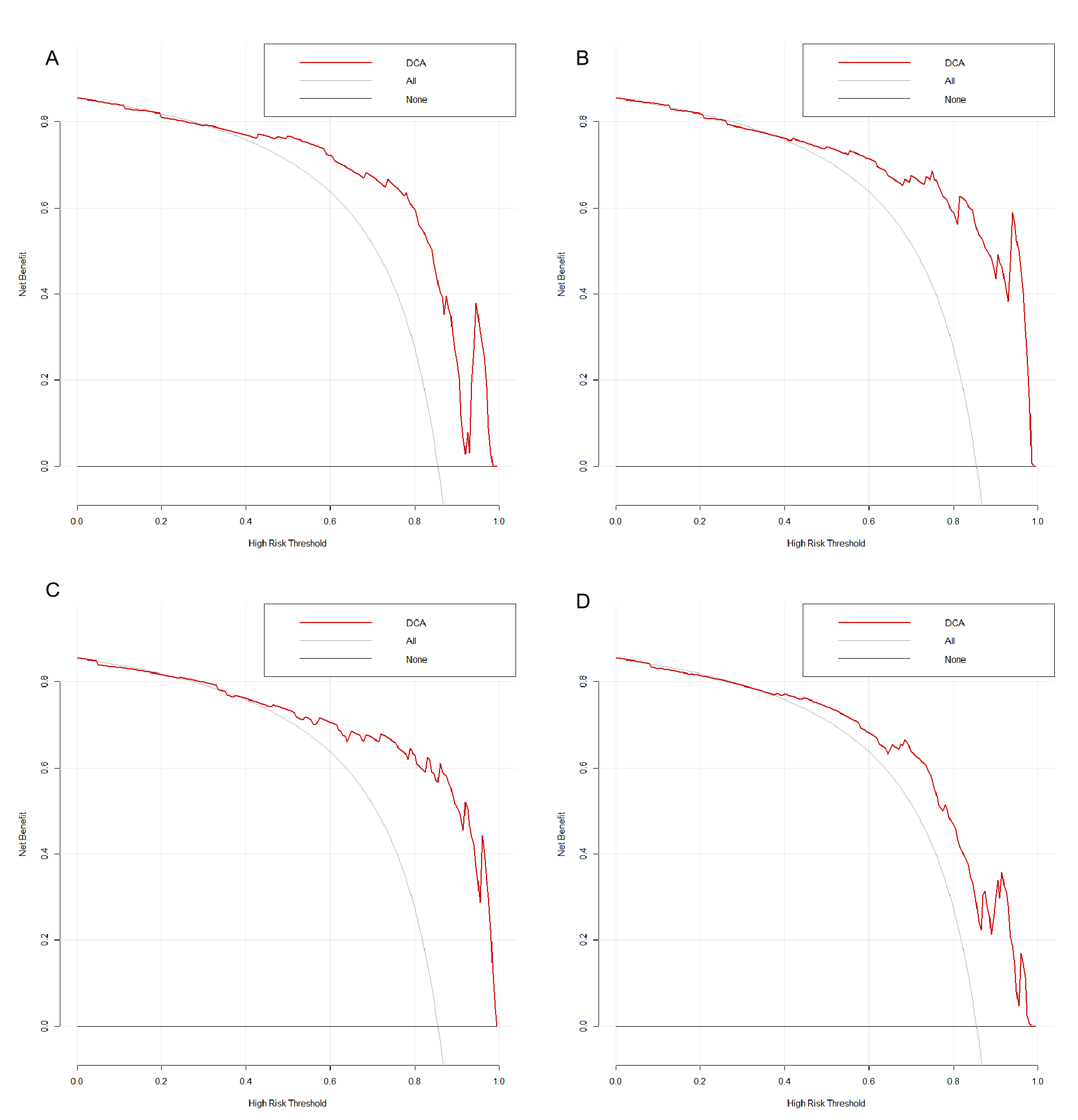

Supplement: Supplementary Figure 4 — Decision curve analysis plots of 4 AutoML models in the test set, indicating net benefits of around 80%. (A) DL model; (B) GBM model; (C) GLM model; (D) DRF model. [file Image_4.tif]
