# Supplementary material for: Automated machine learning to predict the difficulty for endoscopic resection of gastric gastrointestinal stromal tumor
Source: Front Oncol. 2023 May 10;13:1190987. doi: 10.3389/fonc.2023.1190987 (PMC10206233; doi:10.3389/fonc.2023.1190987)
Supplement: Supplementary file 5 [file Table_1.docx]

**Supplementary Table 1.** Comparison of AutoML models and logistic regression analysis in predicting the difficulty for ER of gGISTs in the test cohort

|  | **AUC** | **Sensitivity** | **Specificity** | **Accuracy** | **PPV** | **NPV** | **LR+** | **LR-** |
| --- | --- | --- | --- | --- | --- | --- | --- | --- |
| **AutoML** |  |  |  |  |  |  |  |  |
| **GBM** | 0.791 | 0.636 | 0.938 | 0.911 | 0.500 | 0.964 | 10.273 | 0.388 |
| **DL** | 0.782 | 0.625 | 0.926 | 0.887 | 0.556 | 0.943 | 8.438 | 0.405 |
| **DRF** | 0.694 | 1.000 | 0.862 | 0.863 | 0.056 | 1.000 | 7.235 | 0 |
| **GLM** | 0.746 | 0.600 | 0.942 | 0.887 | 0.667 | 0.925 | 10.400 | 0.424 |
| **Logistic regression analysis**  **LASSO** | 0.764 | 0.556 | 0.928 | 0.911 | 0.769 | 0.928 | 19.630 | 0.457 |

ER: endoscopic resection; gGIST: gastric gastrointestinal stromal tumor; AutoML: automated machine learning; PPV: positive predictive value; NPV: negative predictive value; LR+: positive likelihood ration; LR-: negative likelihood ration; AUC: areas under the receiver operating characteristic curves; GBM: gradient boost machine; DL: deep neural net; DRF: default random forest; GLM: generalized linear model; LASSO: least absolute shrinkage and selection operator
